# Hospital Meetings

**Published:** 1896-02-22

**Authors:** 


### HOSPITAL MEETINGS.

### THE ROYAL EAR HOSPITAL, SOHO.

The Royal Ear Hospital, Soho, held its seventy-ninth annual meeting last week. Mr. T. Pallister Young, LL.B., in the absence of the President (the Earl of Dartmouth), presided, and in presenting the annual report, expressed a hope that the hospital would soon be enabled to acquire a building more adapted to its special requirements, and with accommodation for a school of otology worthy of the metropolis. The attendances of out-patients during the past year numbered 8,714, the number of in-patients being 213. The receipts for the year amounted to £757 0s. 5d., and the expenditure was £746 15s. 10d. A cordial vote of thanks was accorded to the honorary medical staff, the hon. auditors, and the hon. treasurer, for their services during the past year. A vote of thanks to the chairman closed the proceedings.

### ROYAL NATIONAL HOSPITAL FOR CONSUMPTION, VENTNOR.

In the absence of Sir Richard Webster (chairman of the board of management), Lord Glenesk presided over the annual general meeting of governors of the Ventnor Hospital for Consumption, held at the London office, 34, Craven Street, Charing Cross, on Thursday, February 13th. Nine or ten governors put in an appearance, amongst whom were the Earl of Feversham, Mr. Neale F. Horne, Admiral de Kantzow, Mr. H. C. Richards, Mr. Savile, and the Rev. the Master of St. Katharine's.

The business before the meeting consisted simply in the re-election of members of the board of management and various officers, and the passing of the annual report. Financially, the hospital maintains a very satisfactory condition, the total receipts in 1895 amounting to £15,133 (including legacies, £4,623), and the expenditure to £11,160. A portion only of a bequest from Mrs. Emily Jane Mullett of £5,000 (less £500 legacy duty) was invested, the remainder being used to meet expenditure. This was somewhat less than in the previous year, and the opportunity had been taken to erect a new engineer's house, and to improve arrangements at the entrance lodge for better supervision. It will be remembered that this hospital is built on the separate principle, and consists of ten blocks of houses with accommodation for 134 men and women patients, each patient having a separate bed-room facing south and overlooking the sea. More room is needed, for not only is the hospital always full, but there are at the present time 150 applicants waiting admission. Mr. Neale Horne, the deputy chairman, stated that vigorous steps would be taken to erect another block during the present year, and it was hoped that charitably-disposed people would undertake to build the houses in memory of relatives or friends, as had been done in the past in the case of the other blocks. Mr. H. C. Richards suggested that as the late Prince Henry of Battenberg had been much interested in the hospital, the inhabitants of the Isle of Wight might subscribe to provide one of the houses in his memory.

The medical report stated that the cases admitted during 1895 numbered 778, some of whom were resident as long as thirty weeks, though the average stay was nine weeks. Of these, 509 were improved in health by the treatment. The cases on the whole had been of a more severe character than usual.

It was announced that the festival dinner in aid of the funds of the hospital would be held on April 20th at the Hotel Metropole, when the Attorney-General would preside.

### LONDON FEVER HOSPITAL.

Lord Balfour of Burleigh presided over the annual meeting of governors of the London Fever Hospital, Liverpool Road, held at the Hotel Victoria, Northumberland Avenue, on Friday, February 14th. The report laid before the meeting stated that a complete rebuilding of the hospital had been decided upon, and also the establishment of a home in connection with it, to which scarlet fever patients might be sent during convalescence. For this purpose a site had been purchased of about nine acres in the north of London, some seven miles from the hospital, and the committee trusted they might receive sufficient funds to enable them to begin the building during the present year. The medical report stated that 516 cases, the majority being scarlet fever, were admitted in 1895, and 446 patients were discharged cured during the same period. The report was adopted, and Lord Balfour of Burleigh, the president, the vice-presidents, treasurer, and other officers were re-elected.

### ST. JOHN'S HOSPITAL FOR DISEASES OF THE SKIN.

Opening of Branch Hospital.

On Monday last Arlington House, Uxbridge Road, was opened by the Countess of Chesterfield for the reception of in-patients from the Leicester Square Hospital. Arlington House is an ordinary detached suburban building, standing in a small garden, about five minutes' walk from Uxbridge Road Station. It is intended to maintain forty beds here, reserving only twelve for urgent cases at the hospital in Leicester Square, where the out-patients' department will continue to be worked as usual. The new house has been taken on lease, and certain alterations and additions have been carried out to render it more fit for hospital purposes. Quite an imposing array of Church dignitaries took part in Monday's proceedings; the Bishop of Marlborough conducting the dedicatory services, held in one of the wards, and the Archdeacon of Middlesex and the Rev. E. G. Wood were also present, Cardinal Vaughan arriving rather late. The Earl of Chesterfield, who is the president of St. John's Hospital, gave a brief summary of its history, and explained that want of proper accommodation had made some extension absolutely necessary. Lady Chesterfield then declared the hospital open, and after rather long-drawn-out votes of thanks the audience found their way into the tea-room and to inspect the various wards, which looked very bright and comfortable with their rows of neat beds. It is, however, never possible to convert an ordinary dwelling house into a proper hospital, and the placing of the ward for men close to the kitchen and in the basement is anything but satisfactory. The rooms for the nurses, too, are inadequate, the four nurses sleeping in two rooms each of which is none too large for one person. The matron, Miss Newberry, whose red uniform by the bye looked particularly cheerful and pretty, has a pleasant bed and sitting-room allotted to her. There is one ward for children and two for women, besides that for men already mentioned, and a pay ward of two beds.

### ST. GEORGE'S AND ST. JAMES' DISPENSARY.

The Hon. W. F. D. Smith, M.P., presided at the festival dinner of the above, which was held at the Whitehall Rooms on the 18th inst. Among those present were Sir A. N. Birch, Sir Clement Hill, Dr. Godson, the Rev. P. T. Bainbridge, the Treasurer (Mr. W. G. J. Marjoribanks), and the Secretary (Mr. St. Leger Bunnett). The Chairman pointed out that this dispensary, which was established in 1817, had experienced the lot of numbers of the older charities in that many of their old supporters had passed away, and the needs of the newer charities had been so prominently brought before the public that the younger supporters of charities had overlooked its necessities. Situated as it was in an extremely important district, and in a district which one would have thought would have been willing to support a charity of such public use, it yet was in want of funds, as its income from annual subscriptions amounted to but £185, against an expenditure of £550 a year. The total annual income of the charity was only £310, so that the invested funds of the institution have had to be drawn on for about £240 a year. This had reduced the funds of the institution to almost nothing. Since its establishment over 464,000 patients had been treated, of whom 5,700 odd were treated during 1894. This showed that, without doubt, if the dispensary ceased to exist its loss would be very much felt among the poor of the district, for in that case a very large number of patients would have to be transferred to other charitable institutions in the neighbourhood, or else those patients would when sick have to seek help in another district. The Chairman said he had been through the accounts of the institution, and had been unable to find any items which could be cut down. If, he said, it could be brought home to the inhabitants of the district of St. James' that so extraordinarily useful an institution was on the verge of being closed for want of funds its needs would, he was sure, be promptly met. The dinner resulted in a gain to the institution of £400, of which over £70 was in annual subscriptions. A point which is very often overlooked in the work of this dispensary is that a very large proportion of its patients are drawn from the tailoring hands who work for the West-end tailors. The work that this institution does should, therefore, appeal to all living in the West-end, as there is no doubt that by its means diseases of an infectious character are prevented from spreading to the houses of the richer inhabitants of the district.

